# Alcohol and Tobacco Use During the COVID-19 Pandemic. A Call for Local Actions for Global Impact

**DOI:** 10.3389/fpsyt.2021.634254

**Published:** 2021-02-18

**Authors:** Rodrigo Ramalho, Frances Adiukwu, Drita Gashi Bytyçi, Samer El Hayek, Jairo M. Gonzalez-Diaz, Amine Larnaout, Laura Orsolini, Victor Pereira-Sanchez, Mariana Pinto da Costa, Ramdas Ransing, Mohammadreza Shalbafan, Zulvia Syarif, Paolo Grandinetti

**Affiliations:** ^1^Department of Social and Community Health, School of Population Health, University of Auckland, Auckland, New Zealand; ^2^Department of Neuropsychiatry, University of Port Harcourt Teaching Hospital, Port Harcourt, Nigeria; ^3^Community Based Mental Health Center and House for Integration Prizren, Hospital and University Clinical Service of Kosovo, Prizren, Kosovo; ^4^Department of Psychiatry, American University of Beirut, Beirut, Lebanon; ^5^CERSAME, School of Medicine and Health Sciences, Universidad del Rosario - Clínica Nuestra Señora de la Paz, Bogotá, Colombia; ^6^Department of Psychiatry, Faculty of Medicine of Tunis, Razi Hospital, Tunis El Manar University, Tunis, Tunisia; ^7^Unit of Clinical Psychiatry, Department of Neurosciences/DIMSC, Polytechnic University of Marche, Ancona, Italy; ^8^Department of Child and Adolescent Psychiatry, NYU Grossman School of Medicine, New York, NY, United States; ^9^Unit for Social and Community Psychiatry, Queen Mary University of London, London, United Kingdom; ^10^Institute of Biomedical Sciences Abel Salazar, University of Porto, Porto, Portugal; ^11^Hospital de Magalhães Lemos, Porto, Portugal; ^12^Department of Psychiatry, BKL Walalwalkar Rural Medical College, Kasarwadi, India; ^13^Mental Health Research Center, Department of Psychiatry, School of Medicine, Iran University of Medical Sciences, Tehran, Iran; ^14^Department of Psychiatry, Tarakan General Hospital, Jakarta, Indonesia; ^15^Addictions Services (SerD), Department of Territorial Assistance, ASL Teramo, Teramo, Italy

**Keywords:** COVID-19, pandemic, public health, alcohol, tobacco, alcohol policy, tobacco control

Faced with the COVID-19 pandemic, the world was forced to adopt strong public health measures, such as travel restrictions, physical distancing, and self-isolation. Prolonged periods of self-isolation, like the one imposed by the ongoing pandemic, may have serious repercussions on people's mental health (1, 2). For example, these restrictive measures could potentially lead to an increase in the incidence of risky behaviors, like smoking or excessive alcohol use and medical conditions due to increasing smoking and alcohol use, as well as an increased risk of domestic violence (3–9). Furthermore, harmful patterns of substance use, including hazardous patterns of drinking and smoking, represent a risk during lockdowns due to the prolonged periods of self-isolation necessary to control the transmission of the virus (1, 10, 11). Unfortunately, despite ongoing research efforts, there is still sparse information about the impact of the COVID-19 pandemic on substance use patterns.

We conducted a search on August 8th, 2020 for peer-reviewed publications in English using three databases: PubMed, ProQuest, and Web of Science. We searched for publications that had the following keywords in their titles: “alcohol” or “drinking” or “smoking” or “nicotine” or “cigarette” or “cigarettes” or “cigar” or “cigars” and “COVID-19” or “pandemic” or “SARS-CoV-2,” a search that led to ~300 publications. We found two publications regarding potential changes in tobacco use patterns due to the pandemic in the general population. One, a study describing a survey conducted in the United States of America (USA), where almost half of the respondents reported no changes in their smoking patterns, and about a quarter reported having reduced their cigarette smoking (12). There was also a study reporting an increase in tobacco quit attempts during COVID-19 in Italy, India, South Africa, the United Kingdom (UK), and the USA (13), with similar findings being reported in Turkey (14).

Regarding alcohol use, we found a publication reporting an increase in alcohol sales during the early stages of the pandemic in the UK (15). We also found a report of a higher rate of harmful alcohol consumption in the province of Hubei, China (16), a report of a higher rate of alcohol use associated with COVID-19 related psychological distress in the USA (17), and one of increased alcohol use following the closure of a university campus in Ohio, USA (18). We also found two studies reporting an increase in alcohol withdrawal syndrome in India, following lockdown measures (19, 20). In a survey conducted in Germany, 35% of respondents reported consuming more alcohol during lockdown (vs. ~38% who reported no changes) (21); and one in Poland found that 14% of the respondents used more alcohol during the COVID-19 pandemic (vs. 16% who reported drinking less) (22).

To complement this search, the authors also looked for country-specific information regarding restrictions on alcohol and tobacco sales, if there were any, and changes in patterns of alcohol and tobacco use in their respective countries. To conduct this search, the first and last authors invited fellow mental health professionals, members of a team connected through the World Psychiatric Association (WPA) (23), to share information related to their country. The resulting team comprised members from a diverse range of countries. These countries included lower-middle-income (India, Nigeria, Indonesia, and Tunisia), upper-middle-income (Colombia, Lebanon, Iran) and high-income countries (Italy, USA), plus one non-United Nations' country, Kosovo. Official reports and literature review of emerging knowledge about COVID-19 and its impact on these issues were the preferred data source. However, the scarcity of information retrieved from those sources made it necessary for the team also to resort to local media outlets, polls, and anecdotal evidence portrayed in the media.

Restrictions on alcohol sales as a response to the pandemic vary among countries represented in our team, within a continuum that goes from total alcohol ban to no restrictions besides those caused by physical distancing. In India, for example, there was a nationwide alcohol ban during initial stages of lockdown (24), which seemed to have led to an increased incidence of cases of alcohol withdrawal syndrome during that time (19, 20). In later stages, some states, like Delhi, implemented a “special corona fee” on all categories of liquor, a fee currently withdrawn (25). Iran had banned the marketing and consumption of alcoholic beverages decades before the pandemic (26). However, rumors about alcohol consumption as a protective factor against the virus were reported to have led to more than 700 deaths due to methanol intoxication in that country, a common adverse event that follows drinking homemade contaminated alcoholic beverages (27–29). In Tunisia, a few local governors closed liquor stores in their regions (30).

In most countries, however, even during stricter lockdown periods, alcohol sales have been allowed in liquor stores, supermarkets, and retailers. In Nigeria, alcoholic beverages are considered essential commodities, with liquor stores exempted from the lockdown (31), despite the closing down of bars and clubs. Similarly, in the USA, liquor stores were considered essential businesses and they remained open during the times of stricter lockdown (except for the state of Pennsylvania). A survey published in early April showed that drinking had increased in some populations in the USA, including people with previous hazardous drinking patterns (32); also, there are reports of an overall increase in alcohol sales nationwide (33). In Colombia, an online survey reported alcohol to be the second most consumed substance during the COVID-19 related quarantine, after cannabis (34). In Italy, and apparently facing a rise in alcohol consumption, health officials published a report debunking some misinformation about alcohol use as a protective factor against the virus (35). On the other hand, there seems to be a reduction in alcohol sales and consumption in Indonesia (36).

There have been no restrictions on tobacco sales in any of the contributing authors' countries. However, in Colombia, cigar shops can only remain open as long as they also distribute food and basic necessities. In India and the USA, accessibility to tobacco via retailers has varied across states. In the USA, there were reports of tobacco sale increases (13, 37). An increase in tobacco use at home was reported in Italy and India (13). Still, a survey conducted by Yach (13) reported a rise in tobacco quit attempts in the USA, India, and Italy. In Colombia, a recent survey reported that 8% of the respondents have experimented with tobacco for the first time during the pandemic (34). In Indonesia (38), tobacco use has decreased during the lockdown, while a report in March suggests the same happening in Tunisia (39).

Our findings concur with the suggestions made by other authors that, during the COVID-19 pandemic, tobacco and alcohol use patterns have been influenced by societal and cultural processes, as well as by local alcohol control policies (40, 41). We found various factors potentially playing a role in a country's trend of alcohol and tobacco use during the current pandemic besides public health and trading policies, such as public health campaigns and misinformation, socioeconomic conditions, cultural background, and the prevalence of substance use disorder or psychological distress.

The pandemic has led the world to recognize the need for global action in order to support people's health and well-being. It is necessary for all countries to develop measures that will support the entire population during this time of crisis, including people with a substance use disorder. These measures should incorporate effective demand, supply, and harm reduction strategies to reduce risky substance use and substance-related harm. In regard to alcohol and tobacco, potential ways forward include revising local alcohol and tobacco licensing systems and reducing hours of sale, reducing availability via carry out and delivery services, promoting help seeking and reducing stigma around it, providing sustained public health promotion campaigns, and fostering diversion initiatives that could be conducted while observing physical distancing. It is of the utmost importance for any strategy to be evidence informed, locally relevant, culturally appropriate, and equitable. In other words, it is relevant and necessary local actions that would lead to global impact, and the time for action is now.

## Author Contributions

RRam and PG developed the concept of this manuscript and discussed it with FA, DG, SE, JG-D, AL, LO, VP-S, MPC, RRan, MS, and ZS. FA, DG, SE, JG-D, AL, LO, VP-S, RRan, MS, ZS, and PG provided country-specific information. RRam and PG wrote the initial draft and FA, DG, SE, JG-D, AL, LO, VP-S, MPC, RRan, MS, and ZS edited and approved the final version for submission. All authors contributed to the article and approved the submitted version.

## Conflict of Interest

The authors declare that the research was conducted in the absence of any commercial or financial relationships that could be construed as a potential conflict of interest.
